# On the Action of Medicines

**Published:** 1848-01

**Authors:** 


*On the Action of Medicines.*—By James Blake, M. D., Professor of Surgery in the St. Louis University.

Having been engaged for some years in an experimental investigation of the action of medicines on the animal economy, I propose to lay before the medical profession a *resume* of the results of my inquires, not so much with the object of affording any direct information on the therapeutical effects of our various medicinal agents, as to direct attention to some physiological phenomena, which seem to indicate the laws that govern the reactions between living beings and inorganic compounds.

In the present state of physiological science, it is not to be expected that all the indications it affords, can meet with their direct application in pathology, for this would be supposing it to have attained a much greater state of perfection than even its most ardent admirers can claim for it; but, notwithstanding this apparent separation between physiology, and pathology and therapeutics, I am perfectly convinced that the progress of medicine, as a science, must depend on the advancement of our physiological knowledge. In the remarks I have to make, I shall endeavor to throw some light on the most important and fundamental questions connected with the subject of the action of medicines, viz: as to whether medicines act by being taken into the circulation, or whether they produce their effects by impressions made on the nerves of the part to which they are applied, which impressions are capable of destroying or strongly modifying the functions of the nervous centres, and by this means reacting on the whole system? Another question which I have submitted to the test of an experimental examination is,—when a substance is absorbed into the blood, do the reactions it gives rise to, depend on the chemical properties of the substance used? or, in other words, are the reactions between the elements of the blood and tissues, and inorganic compounds, governed by the same laws when taking place in living animals, as they are when these same elements are mixed together out of the body, and no longer forming a part of the living organism? It is evident that the solution of these important questions must precede all further scientific investigation on the action of medicines, and yet, on both these points, the opinions of physiologists and pathologists are still undecided; for, whilst some admit to its fullest extent the doctrines of nervous sympathy, and the sudden annihilation of all the powers of the nervous system by the impressions made on the peripheral portions of the system,—others, on the other hand, consider that it is only when a substance has been absorbed, that it is capable of producing any marked effects in modifying or destroying the functions of this system. There is, also, the same want of unity in the opinions of physiologists as to the laws which govern the reactions between inorganic compounds when once taken into the blood, and the elements of the living being with which they are thus brought into contact, some viewing the changes that take place under these circumstances as the result of the ordinary chemical properties of the substances used, whilst others, on the contrary, adopt the opinion that the moment a substance is brought into contact with the elements of a living body, its common chemical properties cease to be called into play, or are overpowered by some property, the existence of which they have imagined under the name of vitality. I trust the importance of these questions will be a sufficient excuse for my entering somewhat into detail on the experiments which I have undertaken to elucidate them. Although these experiments have been published, from time to time, in some of the European medical journals, yet I shall not hesitate to again bring them forward, as the journals in which they have appeared are not accessible to many of your readers, and also, that the detached form in which they have been published must necessarily tend to weaken the evidence they afford in elucidating the object of these researches.

The first and most fundameutal question which presents itself in entering on the investigation of the action of medicines, is that which relates to their absorption. As I have before stated, physiologists are not yet agreed on this point, some being of opinion that many substances can act powerfully on the system without being taken into the blood,—whilst others affirm that it is only after these substances have entered into the blood vessels and have been carried to the organs whose functions they modify, that they can produce any effects. Amongst the supporters of the former opinion are Valentin, Bostock, Carpenter, Christison, and many others; whilst Magendie, Muller, Dunglinson, and others, think that before a substance can produce any general effects on the economy, it must previously have been taken up by the blood vessels, or absorbents, and have become applied directly to the organ affected. The principal arguments brought forward in support of the former opinion, are the rapidity with which certain poisons are known to produce their effects when applied to the eye, or other mucous surfaces, it being argued that a sufficient time does not elapse between the application of the poison and the first symptoms of its action, for it to have been taken into the blood and carried to the central parts of the nervous system. It has also been stated, that when the blood of a poisoned animal is made to circulate through the brain of another animal, no effects are produced on the second animal, although such should be the case, had the blood been the medium through which the poison acted. It is evident, that the first of these arguments, if founded on fact, must be fatal to the theory that poisons are necessarily absorbed before they produce their effects, for if it can be shown that a substance can act on any organ in a shorter time, than would be required for it to be carried to that organ through the medium of the circulation, it is evident that it must act by some other means than by its contact with the organ.* A superficial consideration of the question would at once lead to the rejection of the theory of the absorption of poisons, for it has been stated, and is, I think, generally believed, that some of our most rapidly fatal poisons, such as hydrocyanic acid, conia, and woorara, produce their effects so rapidly that no appreciable interval elapses between their application to the eye or the mucous membranes, and the appearance of the first symptoms of their action on the nervous system. A carefully conducted series of experiments, however, has convinced me that an interval, which is always appreciable, elapses between the application of a poison to a mucous surface, and the appearance of the first symptoms of action, and that this interval varies in different animals. As this is a point, which it is of much importance to establish, I shall relate some of the experiments that have been performed with the view of elucidating it.

The substance with which the greatest number of experiments have been tried, and which is supposed to produce instantaneous effects,

* The hypothesis by which Muller has attempted to set aside this inference seems to me inadmissable. Wishing to reconcile the rapidity of the action of poisons, with his ideas of the velocity of the circulation, he considers, that although some poisons produce their effects in a shorter time than they could arrrive at the nervous centres through the medium of the blood, yet in these instances the poison reaches the brain by directly permeating the tissues that are interposed between the brain and the part on which the poison is applied.

when applied even in small quantities to a mucous surface, is the concentrated hydrocyanic acid. It is stated, that a drop or two applied to the conjuctiva of a dog, will instantaneously destroy life. To test this assertion, I prepared half a drachm of the anhydrous acid by passing dried sulphuretted hydrogen over bicyanide of mercury.* The whole ot this acid was poured on the tongue of a large dog, the head being kept elevated and the mouth held open: no symptoms of the action of the poison appeared until eleven seconds had elapsed; the respiration then became affected, and the animal was dead in thirty seconds from the time in which the poison was poured on the tongue.

If a tube is introduced into the trachea, a longer interval elapses between the application of the poison to the mouth. and the appearance of the first symptoms of its action; this is owing, undoubtedly, to the poison in this case having a longer distance to go before reaching the brain, for owing to the great volatility of the strong hydrocyanic acid, it is instantly converted into a vapor on coming into contact with so warm a surface as the mouth; it thus becomes inhaled into the lungs, and is there absorbed by the mucous surfaces, and reaches the brain through the pulmonary veins, the left cavities of the heart, the aorta and carotids; whereas, when it is prevented from entering directly into the lungs, it has, in addition to the above course, to pass down the jugular vein, into the right side of the heart, and through the pulmonary artery. A tube was introduced into the trachea of a dog, through which respiration was carried on: a drachm of concentrated hydrocyanic acid was poured on the tongue of the animal, and no symptoms of its action appeared until sixteen seconds elapsed; respiration then became affected, the animal was strongly convulsed, and died about forty-five seconds after the application of the poison. These experiments have been frequently repeated and always with the same results; they show, that at least, as regards hydrocyanic acid, and appreciable interval elapses between the administration of the poison and the appearance of the first symptoms of its action, and although this interval might appear scarcely worth recording, and has, from its shortness, probably escaped the observation of former experimenters in the confusion and hurry of the experiment, yet it will be seen from the subsequent part of this paper, that it forms a most important element in determining the question which we are now discussing.†

* In order to insure a marked effect, the whole of the acid was used, although a simple drop would have been enough to have killed the animal.

† I would here caution any of your readers, who may wish to repeat these experiments, against an accident which is very likely to happen, whilst experimenting with such large quantities of so volatile a poison as hydrocyanic acid. As I before observed, the acid is converted into vapor the instant it comes into contact with the surface of the mouth, and it is present in large quantities in the air, expired by the animal, so as to render this highly poisonous, if inhaled into the lungs of a person who might be leaning over it, or holding open the mouth. I was almost poisoned from this cause, whilst conducting some of these experiments, the effects of the poison being felt for many days.

The next experiments I shall relate, are those which have been performed with conia, the active principle of the hemlock. Dr. Christison has published a series of experiments, apparently conducted with great care, which he performed with this substance, and as the results he has arrived at, differ from those which I have to bring forward, I have endeavored to avoid as much as possible every source of error, and by frequently repeating the experiments, to satisfy myself that the discrepancy in our results could not be owing to any want of care on my part.

I obtained a considerable quantity of conia from an alcoholic extract of the seeds of conium maculatum, which, in its physical and chemical properties, exactly resembled that described by Dr. Christison. As this substance is not so readily absorbed as the hydrocyanic acid, the best method of observing its effects is to introduce it directly into a vein. Ten drops of the poison, (one drop would have been amply sufficient to have produced death,) rendered soluble by a small portion of hydrochloric acid, were injected into the femoral vein of a dog; not the slightest symptom of the action of the poison manifested itself until fifteen seconds after its introduction into the veins. The animal then became convulsed, and died in thirty seconds after the administration of the poison; thus affording a marked proof of its virulence, for by no other substance that I have experimented with, has death been produced in so short a time.* This experiment has been repeated four times, and in no instance have any symptoms appeared in less than fifteen seconds.

The woorara is a poison which has been stated to produce instantaneous death. Having obtained a quantity of this substance from Sir B. Brodie, I proceeded to test its powers: five grains of the poison, dissolved in water, were injected into the jugular vein of a dog; no symptoms of the action of the poison showed themselves until twenty seconds after it had been injected. Violent tetanic convulsions then came on, and the animal died about forty-five seconds after the injection.

The striking effects produced by nux vomica, or its active principle, strychnia, on the nervous system, are such as to enable us accurately to seize the first symptoms of its action, and for this reason I have used it very extensively in these experiments. A solution containing five grains of strychnia, was introduced into the jugular vein of a dog; spasms of the muscles were observed twelve seconds after the introduction of the poison, and the animal died in about a minute and half. The facts contained in the above experiments, together with many others that I could bring forward, go to prove, that even with the most rapidly fatal poisons, an interval always elapses between the application of a poison to any

---

* The reader will perceive by preceding and following experiments that this statement is erroneous.—Ed. Buff. Jour.

part of the body, and the appearance of the first symptoms of its action; and therefore, that the evidence which the consideration of this part of the subject furnishes, does not lend any support to the opinion that poisons act by a direct impression produced in the nerves of the part to which they are applied.

I shall now proceed to relate some experiments which have been performed on another point, closely bearing on this subject. As it is well known that the rapidity of the circulation differs considerably in different classes of animals, it is evident, that if a poison acts by being carried to the nervous centres, through the medium of the circulating fluid, it must reach the brain most rapidly in those animals in which the circulation is quickest, and therefore we should expect to find an appreciable difference between the time required for the poison to act in a larger or smaller animal—or according as the circulation was completed in a longer or shorter period. In order to ascertain, if this were the case, I have made experiments on the horse, the dog, the rabbit, the fowl, and the goose. In these comparative experiments, I have generally made use of strychnia or woorara, on account of the first symptoms of their action being so readily perceived, that they seem to be the most rapid of all poisons, in producing their effects.

A solution containing six grains of strychnia, dissolved in about three ounces of water, slightly acidulated, was introduced into the jugular vein of a strong, healthy horse. The animal was not confined, so that the first symptoms of the action of the poison could be well observed; sixteen seconds after the injection of the poison, the first symptoms of its action showed themselves by slight twitchings of the muscles of the thorax; seventeen seconds after the injection of the poison, the animal fell, as if it had been a mass of marble, every muscle in the body being in a state of the greatest rigidity; this lasted for about a minute and a half. Tetanic spasms then came on, and lasted till the animal died, about four minutes after the injection of the poison.

When strong doses of the concentrated hydrocyanic acid are poured on the tongue of a horse, the poison does not show any symptoms of its action until after an interval of from twelve to seventeen seconds.

The time required for these poisons to act, when introduced into the veins, or applied to the mouth of the dog, has already been stated to be from eleven to twelve seconds, an interval four or five seconds shorter than the time required for them to produce any effect upon the horse.

On the fowl, the interval is still much shorter. A grain and a half of nitrate of strychnina, dissolved in a small quantity of water, was introduced into the jugular vein of a fowl; in six and a half seconds after the introduction of the poison into the vein, the animal became convulsed, and so rapid was the action of the poison, that in eight seconds the animal lay, to all appearance, dead with the exception of slight movements of the tail.

In the rabbit, again, the interval that elapses between the introduction of a poison into the veins and the appearance of the first signs of action, is still shorter than in the fowl; so rapid, in fact is the action of poisons on this class of animals, that it is not surprising that the interval that occurs between the application of the poison and the first symptoms of its action, has been altogether overlooked; and as experiments with poisons are most generally performed on these animals, this has tended to favor the idea of their instantaneous action.

A solution, containing half a grain of nitrate of strychnia, was injected into the jugular vein of a full grown rabbit; in four seconds and a half, the first symptoms of the action of the poison showed themselves, and in seven seconds after its introduction into the vein, the animal was dead. I have seen the concentrated hydrocyanic acid act in less than four seconds, when it has been poured on the tongue of a rabbit—and it is not surprising, that unless the greatest care is taken in observing the time, so short an interval should have been overlooked. These experiments on different animals have been frequently repeated, and always with the same results. It would thus appear, that in different animals, the interval varies, that elapses between the time when a poison is applied to the tissues, or mixed with the blood, and the appearance of the first symptoms of its action. In the horse, for instance, this period is about sixteen seconds; in the rabbit, four and a half seconds. Did poisons act by an impression made on the nerves of the part to which they are applied, we should expect to find, that they should act with equal rapidity on all animals, as a nervous impression cannot be conceived as lingering on its way to the brain any more in the horse than in the rabbit.

On the supposition that the class of poisons, whose action we are now considering, only act when they arrive at the nervous centres, it is evident that the nearer to these centres is the part where the poison is applied, the more rapid mnst be its action. If, for instance, a poison is injected into the carotid artery, it has evidently a much shorter distance to go before reaching the brain, than when it is injected into the jugular vein.

The following experiment was performed in order to ascertain if this was the case: A tube was inserted into the carotid artery of a horse, the point of the tube looking towards the brain; a solution, containing four grains of strychnia, was injected into the artery. In eight seconds the action of the poison was distinctly visible, by the muscles of respiration being affected; the animal then fell, and expired in about three minutes.

On injecting a solution, containing five grains of woorara, into the axillary artery of a dog, the tube being so inserted that the injection should pass into the aorta, and thus become mixed with the blood, as it was escaping from the heart, to be diffused over the whole system, the first symptoms of the action of the poison showed themselves in seven seconds after its injection, and the animal was dead in thirty seconds.

These experiments have been repeated, and have constantly furnished the same results. It would thus appear, from experiments already related, that, *first*, an *appreciable interval* always elapses between the administration of a poison and the appearance of any symptoms of its action. *Secondly*, that this interval varies in different animals, being shorter and longer, according to the size of the animal and the rapidity of the circulation; and, *thirdly*, that the nearer the point, at which the poison is introduced into the system, is to the brain, the more rapidly are its effects manifested. But a question here presents itself, on the solution of which must depend our admitting or rejecting the doctrine of the absorption of the poison, and its direct application to the organ whose functions are affected. It has been shown, that the interval that elapses between the introduction of a poison into the jugular vein and the appearance of the first symptoms of its action, does not exceed, in the horse, sixteen seconds; in the dog, twelve seconds; in the fowl, six seconds, and in the rabbit, four and a half seconds. Now, it might be asked, do these intervals allow sufficient time for the blood to circulate from the point, where the poison is introduced, to the brain? This is a most important question, and one which it was necessary to determine by experiment; for according to the views which have, until the present day, been entertained, as to the velocity of the circulation, it must appear impossible that a poison can be carried from the jugular vein to the brain, in the horse, in so short a time as sixteen seconds, or in the rabbit in the small interval of four seconds and a half.

The experiments of Herring, on the time required for the blood to circulate in the horse, had shown, that in this animal, the whole round of the circulation might be completed in twenty seconds; but these experiments have not—that I am aware of—been repeated by others, and they certainly have not had that influence on the views of physiologists, on this point, which from their accuracy and importance they deserve.

Herring has shown, that on injecting a solution of prussiate of potassa into the jugular vein of a horse, the salt can be detected in the blood issuing from the vein on the other side of the neck, twenty seconds after its injection into the opposite jugular, so that in this interval the salt must have made the whole round of the circulation. In order to ascertain the velocity of the circulation in the horse, I have performed an experiment analogous to that described by Herring. A tube was introduced into the jugular vein of a strong, healthy horse, another in the carotid artery of the opposite side. In order to avoid any of the injection becoming directly mixed with the blood, the animal was thrown on the side on which the tube was introduced into the jugular, so that the side from which the blood escaped, was the highest, the poison being injected into the vein. A solution, containing one ounce of nitrate of baryta, dissolved in five ounces of warm water, was injected into the jugular vein. Ten seconds after the commencement of the injection, blood was allowed to escape from the carotid, and received into a vessel, No. 1, during five seconds; another vessel was then substituted, No. 2, and received the blood escaping from the carotid, from fifteen to twenty seconds after the introduction of the substance into the jugular vein: what escaped from the artery after this was received into a third vessel, No. 3: the heart had already stopped when the last vessel was applied. These different specimens were carefully analysed; no trace of baryta could be obtained from the blood from No. 1, or that which escaped from the artery between ten and fifteen seconds after the injection of the poison. In the blood from No. 2, baryta was readily detected; and although an accident during the analysis led to the loss of a portion of it, still it yielded a much greater quantity, in proportion to the quantity of blood, than did the contents of No. 3, or the blood obtained from the carotid subsequent to the twenty seconds after the injection of the salt into the veins, thus proving that the blood which was most strongly impregnated with the poison, escaped from the carotid, between fifteen and twenty seconds after the introduction of the salt into the veins. From the large proportion of the salt in the blood that escaped during this interval, we must conclude that the moment at which blood containing the salt first began to flow from the artery, must have been about sixteen seconds after its introduction into the veins. On account of the tube in the carotid being rather small, I think it probable that a second or two might have been lost from this cause, the blood not being able to escape with sufficient freedom.

Another method of measuring the velocity of the circulation, is afforded us by the property which I have discovered some salts to possess, of suddenly arresting the action of the heart, when mixed with the blood circulating over its parieties. The nitrate of potassa is one of these salts, and I therefore availed myself of it to ascertain the time required for the blood to pass from the jugular vein to the capillary terminations of the coronary arteries. A solution containing half an ounce of this salt, was injected into the jugular vein of a horse; in sixteen seconds after the introduction of the salt into the veins, the action of the heart was suddenly suspended, as was shown by the hemadynamometer, an instrument, which, when connected with the arterial system, indicates the slightest change taking place in the circulation.

These two experiments lead to the same results as those of Hering, viz: that in the horse the time required for the blood to complete the whole round of the circulation is about twenty seconds. Analagous experiments have been performed on the dog. In this animal, when a solution of chloride of barium is injected into the jugular vein, it is found to make its appearance in the blood issuing from the carotid artery in about six seconds after its introduction into the vein. A solution of nitrate of posassa, injected into the jugular vein of a dog, arrests the action of the heart in from ten to twelve seconds, so that this interval may be regarded as the time required for the blood to pass, in the dog, from the jugular vein to the capillary terminations of the coronary arteries.

In the fowl, the interval that elapses between the injection of a solution of nitre into the vein, and the arrest of the heart's action, is about six seconds, so that this may be considered as the time required for the blood to pass from the jugular vein to the capillary terminations of the coronary arteries in this class of animals.

On performing analagous experiment on the rabbit, it is found the heart's action is arrested in four seconds and a half after the solution of the salts has been introduced into the jugular vein.

The results of these experiments furnish us clearly with the data that were required to ascertain, if the circulation of the blood takes place with sufficient rapidity to allow of a poison being carried to the brain in the short interval that has been observed to elapse between its application to a mucous surface, or its introduction into the vein, and the appearance of the first symptoms of its action.

On comparing the time, that is required for the blood to complete the round of the circulation, as ascertained in these experiments on different animals, with the time required for a poison to act on these same animals, a most striking agreement will be found between the numbers furnished by the two series of experiments—an agreement so close that it is almost impossible not to admit an intimate connection between the action of poisons and the circulation of the blood. In the horse, for instance, we find that in sixteen seconds the blood will pass from the jugular vein to the capillary terminations of the coronary arteries, and we also find that the same interval elapses between the introduction of a poison into the vein and the appearance of the first symptoms of its action. The same agreement is observed between the rapidity of the circulation in the dog, and the time required for even the strongest poison to produce any sign of its having affected the system.

Even in the fowl, and in the rabbit, in which the actions of poisons is so much more rapid, we find that the circulation of the blood is carried on with proportionable rapidity.

These experiments furnish, I consider, conclusive evidence that no objection can be raised to the theory of the absorption of poisons, on the ground that the circulation is not sufficiently rapid for the blood to carry them to the organs affected, in the time that has been observed to elapse between their application, and the appearance of the first symptoms of their action; in fact, they afford, when considered in their mutual relation to each other, a strong body of negative evidence in favor of the absorption of poisons.

The conclusive evidence that I have adduced as to the interval that elapses between the application of the most rapidly fatal poisons and the appearance of any symptoms of their action, and also the proofs that have been brought forward, (and I could adduce many others if necessary,) of the rapidity of the circulation, will, I hope, be sufficient to modify the opinions of physiologists on these important phenomena. In a future article I shall show that a poision does not produce any effects on the system, although applied extensively to the peripheral extremities of the nerves, unless it is taken into the blood, and thus carried to the nervous centres. I shall offer, also, some critical remarks on the experiments of Addison and Morgan, in which they caused the blood of a poisoned dog to circulate through the carotid of another animal, without producing any morbid symptoms, and I shall then enter on the question of the chemical action of inorganic compounds when taken into the blood.—*St. Louis Med. and Surg. Journal.*

